# Comparison of the Finnish Diabetes Risk Score Model With the Metabolic Syndrome in a Shanghai Population

**DOI:** 10.3389/fendo.2022.725314

**Published:** 2022-02-22

**Authors:** Shenyi Jin, Qingguang Chen, Xu Han, Yahua Liu, Mengjie Cai, Zheng Yao, Hao Lu

**Affiliations:** Department of Endocrinology, Shuguang Hospital Affiliated to Shanghai University of Traditional Chinese Medicine, Shanghai University of Traditional Chinese Medicine, Shanghai, China

**Keywords:** risk predictors, type 2 diabetes, prediabetes, metabolic sydrome, primary screening

## Abstract

**Aims:**

This study aimed to compare the diagnostic accuracy of the metabolic syndrome with the Finnish Diabetes Risk Score (FINDRISC) to screen for type 2 diabetes mellitus (T2DM) in a Shanghai population.

**Methods:**

Participants aged 25-64 years were recruited from a Shanghai population from July 2019 to March 2020. Each participant underwent a standard metabolic work-up, including clinical examination with anthropometry. Glucose status was tested using hemoglobin A1c (HbAlc), 2h-post-load glucose (2hPG), and fasting blood glucose (FBG). The FINDRISC questionnaire and the metabolic syndrome were examined. The performance of the FINDRISC was assessed using the area under the receiver operating characteristic curve (AUC-ROC).

**Results:**

Of the 713 subjects, 9.1% were diagnosed with prediabetes, whereas 5.2% were diagnosed with T2DM. A total of 172 subjects had the metabolic syndrome. A higher FINDRISC score was positively associated with the prevalence of T2DM and the metabolic syndrome. Multivariable linear regression analysis demonstrated that the FINDRISC had a linear regression relationship with 2hPG levels (b’= 036, p < 0.0001). The AUC-ROC of the FINDRISC to identify subjects with T2DM among the total population was 0.708 (95% CI 0.639–0.776), the sensitivity was 44.6%, and the specificity was 90.1%, with 11 as the cut-off point. After adding FBG or 2hPG to the FINDRISC, the AUC-ROC among the total population significantly increased to 0.785 (95% CI 0.671–0.899) and 0.731 (95% CI 0.619–0.843), respectively, while the AUC-ROC among the female group increased to 0.858 (95% CI 0.753–0.964) and 0.823 (95% CI 0.730–0.916), respectively (p < 0.001). The AUC-ROC of the metabolic syndrome to identify subjects with T2DM among the total and female population was 0.805 (95% CI 0.767–0.844) and 0.830 (95% CI 0.788–0.872), respectively, with seven as the cut-off point.

**Conclusions:**

The metabolic syndrome performed better than the FINDRISC model. The metabolic syndrome and the FINDRISC with FBG or 2hPG in a two-step screening model are both efficacious clinical practices for predicting T2DM in a Shanghai population.

## Introduction

The increasing prevalence of diabetes, particularly type 2 diabetes mellitus (T2DM), has reached epidemic proportions worldwide (1). In 2019, 463 million people was estimated to suffer from diabetes, and this number was projected to reach 578 million by 2030, and 700 million by 2045 (2). A recent study (3) indicated that the total number of patients with diabetes in mainland China was approximately 129.8 million (70.4 million men and 59.4 million women). Follow-up studies in China, Finland, and the United States (4) have found that early lifestyle and drug interventions can delay or reduce the risk of developing T2DM by 30–60%. Hence, the identification of these individuals is important and effective (5–8). The responsibility for the care of T2DM patients has been transferred from secondary to primary care in the last two decades, among which prediction tools are crucial for identifying individuals at high risk of T2DM (9).

Insulin resistance (IR), the main indicator used in the diagnosis of T2DM even at certain insulin concentrations, is defined as the decreased glucose uptake by cells. Several methods are available for diagnosing IR (10). The homeostasis model assessment of insulin resistance (HOMA-IR) is one of the most widely used methods for assessing IR. In addition, the homeostasis model for assessing insulin sensitivity (HOMA-IS) and β-cell function (HOMA-β) is always used to observe insulin secretion and pancreatic β-cell functions (11). However, it is necessary to measure serum insulin levels to calculate HOMA, and this measurement is not part of routine evaluation in health care services. In addition, subjects with prediabetes or T2DM are commonly accompanied by a set of risk factors, which is known as the metabolic syndrome. The metabolic syndrome is a cluster of IR and disturbed glucose metabolism, overweight and abdominal fat distribution, dyslipidemia, and high blood pressure (12). Subjects with the metabolic syndrome have a 5-fold higher risk of developing T2DM (13). The metabolic syndrome is present if at least three of the following five criteria are met (14): (1) waist circumference ≥ 102 cm in men or 88 cm in women; (2) blood pressure ≥ 130/85 mmHg; (3) triglyceride (TG) levels ≥ 1.7 mmol/L; (4) high-density lipoprotein cholesterol (HDL-C) levels ≤ 1.03 mg/dl in men or 1.3 mmol/L in women; (5) fasting blood glucose (FBG) ≥ 100 mg/dl. This makes it one of the most labor-intensive and expensive prediction tools for T2DM.

Simple prediction tools that can identify individuals at risk could reduce the cost and inconvenience of screening. With such tools, a two-step procedure could be used: first, subjects would be screened with a risk score, and then those individuals identified to have a high risk for T2DM would have their glycemic status assessed by FBG, 2-h post-load glucose (2hPG) using the oral glucose tolerance test (OGTT), or hemoglobin (Hb)A1c measurements (15). However, due to differences in diet, lifestyle, social environment, and genetic susceptibility of different populations, the applicability of the model is limited (16). Therefore, various regions need to continue to explore T2DM assessment models that are suitable for local populations. The ideal assessment model needs to accurately assess the disease risk of individuals and to identify high-risk groups that are more likely to develop T2DM (discernment), which is a prerequisite for mature risk assessment models (17).

The Finnish Diabetes Risk Score (FINDRISC) model is the most authoritative and widely used risk-scoring model in Europe and other populations (15). The European Society of Cardiology, the European Association for the Study of Diabetes, and the Canadian Preventive Health Care Working Group all recommend the use of the FINDRISC model in the screening of T2DM when formulating the prevention and treatment guidelines for diabetes (15). The original questions in the FINDRISC model included age, body mass index (BMI), waist circumference, physical activity, daily consumption of fruits and vegetables, use of antihypertensive medication, history of high blood glucose, and family history of diabetes. Population-wide screening for diabetes using a standard risk calculator is more acceptable than HbA1c, 2hPG, or FBG, which require invasive testing (17). Many countries, including Finland, Canada, and Thailand, routinely use standard risk calculators to determine who should undergo invasive testing for T2DM diagnosis (17). Receiver operating characteristic (ROC) curve analysis is the most frequently used method to evaluate the discernment of the FINDRISC model (18). The value of the area under the ROC curve (AUC-ROC) is a global summary statistic of the discriminative value of a model, which can be used to illustrate the possibility that scores are higher in individuals with T2DM than in those without T2DM.

Despite the widespread use of FINDRISC models, the discriminatory accuracy of the metabolic syndrome versus the FINDRISC model has not been tested in a Chinese population. Hence, in this study, the metabolic syndrome to the FINDRISC model as a screening tool for T2DM in a Shanghai population was compared.

## Methods

### Source of Data

The study was a cross-sectional clinical study. Patients who underwent physical examination at two community hospitals in Sanlin and Zhangjiang (Shanghai), who were aged between 25 and 64 years, were recruited from July 2019 to March 2020. The target population was stratified and sampled according to age group (every 10 years was 1 segment) and gender to ensure the uniform distribution of all ages and genders. According to the annual incidence of IGT diabetes (8%-11%), the minimum sample size was 470 cases. In this study, 1,000 cases were participated, and finally a total of 713 participants have completed the study. The study protocol was approved by the Ethics Committee of Shuguang Hospital, affiliated with the Shanghai University of Traditional Chinese Medicine (Certificate number 2018-599-28-01). Written informed consent was obtained from all the participants before enrollment. Exclusion criteria included current pregnancy, inability to communicate or stand, previous diagnosis of T2DM, or refusal to participate in the study by not providing informed consent. The participants’ diabetes risk was calculated based on their FINDRISCs.

### Diabetes and Pre−Diabetes Definitions

T2DM was defined using the WHO criteria (19) based on one of the following: FBG ≥ 7.0 mmol/L, 2hPG ≥ 11.1 mmol/L, a random plasma glucose ≥ 11.1 mmol/L, HbA1c ≥ 6.5% (48 mmol/mol) (based on the American Diabetes Association (ADA) criteria); and the criteria for pre-diabetes diagnosis, which is based on impaired fasting glucose (IFG): 6.1 mmol/L ≤ FBG < 7.0 mmol/L or impaired glucose tolerance (IGT): 7.8 ≤ 2hPG < 11.1 mmol/L (20).

### The Metabolic Syndrome Criteria

The metabolic syndrome was evaluated according to the National Cholesterol Education Program Adult Treatment Panel III (NCEP ATP III) (14). Subjects were classified as having the metabolic syndrome if more than three of the following criteria were met:

(1) Waist circumference ≥ 102 cm in men and ≥ 88 cm in women
(2) Hypertriglyceridemia: ≥ 150 mg/dl;
(3) Low-HDL cholesterol: < 40 mg/dl in men and < 50 mg/dl in women
(4) High blood pressure: ≥ 130/85 mmHg or on antihypertensive medication
(5) High FBG: ≥ 100 mg/dl.

### Clinical Evaluation

Participants’ name, age, sex, address, contact number, and history of hypertension, hyperlipidemia, hyperuricemia, and fatty liver disease were recorded in the questionnaire form. Each participant underwent a standard metabolic workup, including a clinical examination with anthropometry. All measurements were performed in the morning, with participants in fasting conditions and the lightest possible clothes. Height was measured to the nearest 0.5 cm using a wall-mounted stadiometer. Body weight was tested using a digital scale to the nearest 0.2 kg with the participants wearing the lightest possible clothes. Body mass index (BMI) was calculated as weight in kilograms over height in m^2^ (21). Waist circumference was measured at the mid-level between the lower rib margin and the iliac crest with the participants in a standing position. Blood pressure was measured on the right arm with the participants in a sitting position. The questionnaire was completed without laboratory tests. The answer to each question was assigned different weighted scores, as shown in **Table 1**. The final score was the sum of the scores from the eight questions, which ranged from 0 to 26.

**Table 1:** Finnish Diabetes Risk Score (FINDRISC) questionnaire.

Item	Standard	Score
Age		
	Under 45 years	0
	45–54 years	2
	55–64 years	3
	>64 years	4
BMI		
	<24 kg/m^2^	0
	24–28 kg/m^2^	1
	>28 kg/m^2^	3
Waist circumference (male)		
	<90cm	0
	90–102 cm	3
	>102 cm	4
Waist circumference (female)		
	<80 cm	0
	80–88 cm	3
	>88 cm	4
Physical Activity ≥ 30 min/d		
	Yes	0
	No	2
Consume fruits and vegetables daily		
	Yes	0
	No	1
History of hypertension		
	Yes	2
	No	0
History of high glucose		
	Yes	5
	No	0
Family history of diabetes		
	No	0
	Yes (non-first-degree relatives)	3
	Yes (first-degree relatives)	5

### Biochemical Variables

Laboratory inspection indicators included FBG, 2-h plasma glucose in the 75 g oral glucose tolerance (2hPG) test, fasting serum insulin level (FINS), HbA1c, triglyceride (TG), total cholesterol (TC), low-density lipoprotein cholesterol (LDL-C), and High-density lipoprotein cholesterol (HDL-C). Insulin resistance and β cell function were estimated using the homeostasis model assessment (HOMA), as described by Song, Manson (11). HOMA-IR was calculated as [FINS (mU/l) × FBG (mmol/L)]/22.5; β-cell function (HOMA%β) was calculated as 20 × (FINS [mU/l]/[FBG (mmol/L) - 3.5] × 100%; insulin sensitivity (HOMA%S) was calculated as 1000/(FINS (mU/l) × (FBG (mmol/L).

Procedures for blood sample collection were explained to the participants by trained laboratory staff. Participants were asked to provide venous blood samples after 8–12 h of fasting. The first blood sample was obtained at the first follow-up after verifying the fasting period. Subsequently, 300 mL of test solution containing 75 g of anhydrous glucose was used as recommended. A new blood sample was obtained after 2 h to measure glucose levels. Blood testing was performed at the Shuguang Hospital Testing Center.

### Statistical Analysis

All categorical variables were summarized as numbers and percentages (%), and the chi-squared test was performed to detect differences between men and women. Continuous variables were expressed as means with standard deviations (SD), and between-group comparisons were conducted using independent sample 2-tailed *t*-tests or Mann-Whitney *U* tests. Pearson correlation analyses were used to measure the association between the variables and the FINDRISCs. Multivariable linear regression analyses were conducted using the FINDRISC as a dependent variable. ROC curves were constructed to show the relationship between the sensitivity and specificity of the FINDRISC for identifying subjects with T2DM. The AUC-ROC was used to evaluate the discriminatory accuracy of the FINDRISC in identifying prediabetes and diabetes subjects in male, female, and overall populations. An AUC-ROC of 1.0 represents a perfect test, with no false positive rate and no false negative rate, while an AUC-ROC of 0.5 indicates that the test performed no better than chance. The cut-off points to identify prediabetes and diabetes were determined by the point with the shortest distance to the upper left corner in the ROC curve, which was calculated as the square root of [(1-sensitivity) + (1-specificity)]. The same statistical analysis was performed to evaluate the discriminatory accuracy of the metabolic syndrome to identify subjects with T2DM. Statistical analyses were performed using SPSS software (version 26.0; Chicago, IL, USA). Statistical significance was set at p < 0.05.

## Results

### Characteristics of the Study Participants

**Table 2** summarizes the basic and clinical characteristics of the 713 participants involved in this study. The mean age of the participants was 45.2 ± 11.2 years (women: 45.1 ± 11.4 years; men: 45.4 ± 10.9 years), and women accounted for 60.6% of the total participants. There was no statistically significant difference between men and women in terms of age (p = 0.655). Men had higher height (168.5 *vs*. 157.7 cm; p < 0.001), waist circumference (83.2 *vs*. 77.9 cm; p < 0.001), weight (68.0 *vs*. 58.4 kg; p < 0.001), diastolic pressure (81.8 *vs*. 78.9 mmHg; p < 0.001), HOMA%S (287.7 *vs*. 286.6; p = 0.016), and TG (1.7 *vs*. 1.3 mmol/L; p = 0.002) values than women. Women had higher FBG (5.1 *vs*. 4.9 mmol/L; p = 0.038), fasting serum insulin (22.7 *vs*. 13.7 mmol/L; p < 0.001), HOMA%β (97.0 *vs*. 96.1%; p = 0.016), and HDL-C (1.4 *vs*. 1.2 mmol/L, p < 0.001) values than men. In addition, women had a stronger family history of high glucose (7.4 *vs*. 4.3%) and diabetes (23.4 *vs*. 18.1%) and were relatively more physically active (69.0 *vs*. 63.7%). With increasing values of basic characteristics in the original FINDRISC questionnaire and increasing levels of lipid metabolic and obesity-related indicators, including systolic and diastolic blood pressure, FBG, 2hPG, HbA1c, fasting serum insulin level, TC, and TG, the FINDRISC values increased significantly. In total, 91% of the participants had a very low to low risk (FINDRISC ≤ 11) of T2DM and 5% had a high or very high risk (FINDRISC ≥ 15) of developing T2DM, according to the FINDRISC scale (**Table 3**). There was no significant difference between men and women in terms of their risk of developing T2DM (p = 0.067).

**Table 2:** Prevalence of components of the modified version of the Finnish Diabetes Risk Score (FINDRISC) by sex.

Characteristics		Female					Male						p
	Total (n = 432)	FINDRISC0–7 (n = 294)	FINDRISC8–10 (n = 70)	FINDRISC11–14 (n = 47)	FINDRISC15–20 (n = 21)	FINDRISC21–26 (n = 0)	Total (n = 281)	FINDRISC0–7 (n = 211)	FINDRISC8–10 (n = 42)	FINDRISC11–14 (n = 18)	FINDRISC15–20 (n = 9)	FINDRISC21–26 (n = 1)	
**Blood tests (±)**													
FBG (mmol/L)	5.1 (1.1)	4.9 (0.7)	5.2 (0.8)	5.9 (1.9)	6.6 (1.9)	0	4.9 (1.1)	4.8 (0.9)	4.9 (0.6)	6.3 (2.9)	5.2 (0.6)	5.0	0.038
2h OGTT (mmol/L)	5.9 (2.6)	5.3 (1.5)	6.3 (2.8)	7.2 (3.8)	9.3 (5.5)	0	5.8 (2.6)	5.5 (2.1)	5.7 (2.1)	8.1 (4.8)	8.1 (2.7)	13.4	0.921
HbA1c (%)	5.8 (0.7)	5.6 (0.5)	5.9 (0.4)	6.2 (1.0)	6.8 (1.3)	0	5.8 (0.7)	5.8 (0.6)	5.7 (0.4)	6.3 (1.4)	6.3 (0.5)	7.6	0.825
Fasting serum insulin, mmol/L	22.7 (26.8)	18.5 (22.4)	27.6 (29.5)	37.0 (33.9)	32.3 (36.5)	0	13.7 (20.9)	11.1 (18.3)	22.2 (22.2)	24.6 (35.6)	12.1 (12.5)	1.4	<0.001
HOMA%β	104.1 (80.9)	102.9 (75.0)	114.5 (98.1)	106.2 (99.4)	83.7 (68.4)	0	87.5 (71.4)	86.1 (71.1)	109.5 (86.1)	80.2 (41.6)	56.6 (24.6)	49.4	0.016
HOMA%S	260.2 (221.0)	262.3 (222.3)	245.3 (221.8)	268.9 (210.8)	259.5 (210.3)	0	320.9 (225.0)	330.0 (226.1)	261.7 (214.2)	270.0 (195.3)	444.2 (198.1)	51.7	0.002
HOMA-IR	1.2 (1.2)	1.1 (1.2)	1.3 (1.4)	1.2 (1.5)	0.9 (0.8)	0	0.9 (1.0)	0.8 (1.0)	1.1 (1.3)	0.8 (0.7)	0.5 (0.6)	1.9	0.003
TC (mmol/L)	4.7 (0.9)	4.5 (0.8)	5.0 (1.0)	4.9 (0.9)	5.2 (1.1)	0	4.6 (0.9)	4.5 (0.9)	5.0 (0.9)	4.8 (0.7)	4.8 (0.8)	6.0	0.491
TG (mmol/L)	1.3 (1.3)	1.1 (0.9)	1.5 (0.9)	1.7 (1.4)	2.9 (3.6)	0	1.7 (2.0)	1.5 (1.5)	2.0 (1.3)	3.1 (4.6)	3.7 (3.7)	2.0	0.002
HDL-C (mmol/L)	1.4 (0.3)	1.4 (0.3)	1.3 (0.3)	1.3 (0.2)	1.1 (0.3)	0	1.2 (0.3)	1.3 (0.3)	1.1 (0.2)	1.2 (0.3)	1.1 (0.2)	1.2	<0.001
LDL-C (mmol/L)	2.5 (0.7)	2.4 (0.6)	2.8 (0.7)	2.7 (0.7)	2.9 (0.7)	0	2.5 (0.7)	2.5 (0.7)	2.9 (0.8)	2.6 (0.6)	2.4 (0.5)	3.3	0.876
**Anthropometrics (±)**													
SBP (mmHg)	123.2 (19.0)	118.9 (17.2)	129.7 (17.2)	134.2 (20.6)	136.9 (22.1)	0	123.2 (15.4)	121.8 (14.7)	126.5 (14.9)	125.2 (19.2)	135.6 (17.3)	130	0.986
DBP (mmHg)	78.9 (10.3)	76.8 (9.7)	81.4 (10.5)	84.9 (8.1)	86.5 (11.2)	0	81.8 (9.7)	80.7 (9.6)	83.3 (9.0)	86.2 (10.9)	88.2 (7.3)	90	<0.001
Height (cm)	157.7 (5.3)	157.9 (5.3)	156.7 (4.9)	157.6 (5.7)	158.4 (5.7)	0	168.5 (5.9)	168.3 (6.1)	168.6 (5.8)	169.1 (3.7)	171.1 (3.5)	163.7	<0.001
Weight (kg)	58.4 (9.4)	55.2 (7.2)	62.3 (9.1)	66.9 (11.0)	70.3 (6.2)	0	68.0 (12.0)	65.1 (11.0)	75.9 (11.3)	75.4 (9.9)	81.6 (7.4)	83	<0.001
**FINDRISC score (±)**	5.8 (4.4)	3.3 (2.3)	8.8 (0.8)	11.9 (1.1)	16.2 (1.5)	0	5.0 (4.1)	3.1 (2.2)	8.6 (0.8)	11.7 (1.1)	16.3 (1.7)	24	0.017
Age, years	45.1 (11.4)	42.5 (11.2)	51.0 (9.8)	50.0 (10.5)	50.0 (7.8)	0	45.4 (10.9)	45.2 (11.1)	44.9 (10.5)	45.7 (9.6)	52.7 (8.2)	55	0.655
Waist circumference, cm	77.9 (10.4)	73.9 (7.8)	83.2 (10.3)	89.1 (9.7)	90.8 (5.9)	0	83.2 (10.0)	80.5 (9.2)	90.6 (7.1)	89.5 (8.1)	96.4 (6.8)	106	<0.001
BMI, kg/m^2^	23.3 (4.0)	22.2 (2.6)	25.3 (3.2)	26.8 (3.4)	28.1 (2.7)	0	23.2 (5.5)	22.9 (3.4)	26.6 (2.9)	26.3 (3.3)	27.8 (1.8)	31.0	0.107
Physical activity ≥ 30 min/d, %	69.0	69.0	72.9	66.0	61.9	0	63.7	71.6	40.5	50.0	22.2	No	0.143
Consume fruits and vegetables daily, %	96.5	96.9	97.1	95.7	90.5	0	92.5	92.4	90.5	94.4	100.0	Yes	<0.001
History of hypertension, %	22.0	10.9	48.6	31.9	66.7	0	24.9	14.7	50.0	50.0	88.9	Yes	0.101
History of high glucose, %	7.4	0.01	0.07	23.4	57.1	0	4.3	0.005	0.024	0.33	0.33	Yes	0.846
Family history of diabetes, %	23.4	12.6	28.6	55.3	85.7	0	18.1	0.06	35.7	77.8	100.0	yes	0.439
**Outcomes (%)**													
Non-T2DM	372 (86)	273 (93)	59 (84)	28 (60)	12 (57)	0	239 (85)	187(89)	38(90)	11 (61)	3(33)	0	0.485
Prediabetes	36 (8)	17 (6)	6 (9)	11 (23)	2 (10)	0	29 (10)	17 (8)	3 (7)	4 (22)	5(56)	0	0.368
T2DM	24 (6)	4 (1)	5 (7)	8 (17)	7 (33)	0	13 (5)	7 (3)	1 (3)	3 (17)	1 (11)	1	0.585
The metabolic syndrome (MS)	110 (25)	33 (11)	32 (46)	29 (62)	16 (76)	0	62 (22)	29 (14)	14 (33)	10 (56)	8 (89)	1	0.300

Data were given by mean (SD). FBG, fasting blood glucose; 2h OGTT, 2-h plasma glucose in the 75 g oral glucose tolerance test; HbA1c, glycated hemoglobin; HOMA%β, homeostasis model assessment of β-cell function; HOMA%S, homeostasis model assessment of insulin sensitivity; HOMA-IR, homeostasis model assessment of insulin resistance; TC, total cholesterol; TG, total triglycerides; HDL-C, high-density lipoprotein cholesterol; LDL-C, low-density lipoprotein cholesterol; H-CRP, hypersensitive C-reactive protein; SBP, Systolic blood pressure; DBP, Diastolic blood pressure; BMI= Body Mass Index, Weight (kg)/height (m)^2^.

**Table 3:** Risk levels of FINDRISC by sex.

		Female			Male			Total		P value
**Risk**	**Score**	**N**	**%**		**N**	**%**		**N**	**%**	**0.067**
**Very low risk**	**< 7**	260	60		197	70		457	64	
**Low risk**	**7–11**	126	29		68	24		194	27	
**Moderate risk**	**12–14**	25	6		6	2		31	4	
**High risk**	**15–20**	20	4.6		9	3		29	4	
**Very high risk**	**≥ 20**	1	0.4		1	1		2	1	

The bold P value (0.067) indicates that there was no significant difference between men and women in terms of their risk of developing T2DM.

According to the ADA diagnostic criteria, 37 participants were newly diagnosed with T2DM, 65 with prediabetes, and 611 participants were diagnosed with non-T2DM. In total, 172 participants met the diagnostic criteria for the metabolic syndrome. The FINDRISC score increased with worsening glucose status, including FBG, 2hPG, and HbA1c. Of the 172 participants who were diagnosed with the metabolic syndrome, 63 had a FINDRISC score ≥ 11.

### Associations Between FINDRISCs and Patient Characteristics

According to the results of the Pearson’s correlation analysis shown in **Table 4**, significant positive correlations between the FINDRISC and the metabolic syndrome were observed, including systolic and diastolic blood pressure (r = 0.34, and 0.300, respectively, p < 0.001), FBG, 2hPG, HbA1c, fasting serum insulin level (r = 0.356, 0.361, 0.358, and 0.219, respectively, p < 0.001), TC, TG, and LDL-C (r = 0.276, 0.281, and 0.238, respectively, p < 0.001). In contrast, there was an inverse association between HDL-C and the FINDRISC (r = -0.211, p < 0.001). Subsequently, a multivariable linear regression analysis was performed using the FINDRISC as an independent variable to reach the final regression model. The p values revealed that the FINDRISC had a significant linear regression relationship with 2hPG (b’ = 0.160, p < 0.0001). According to the standardized coefficient, 2hPG showed the greatest impact on the FINDRISC among all characteristics (R^2^ = 0.527; F = 27.287, p < 0.0001, **Table 5**).

**Table 4:** Pearson’s correlation analysis between FINDRISC and variables.

Variable	Pearson’s coefficient (r)	p value
Height	-0.041	0.281
Weight	0.446	**<0.001**
Systolic blood pressure	0.343	**<0.001**
Diastolic blood pressure	0.300	**<0.001**
FBG	0.356	**<0.001**
2hPG	0.361	**<0.001**
HbA1c	0.358	**<0.001**
Fasting serum insulin	0.219	**<0.001**
HOMA%β	0.040	0.372
HOMA%S	-0.018	0.677
HOMA-IR	0.017	0.701
TC	0.276	**<0.001**
TG	0.281	**<0.001**
HDL-C	-0.211	**<0.001**
LDL-C	0.238	**<0.001**

FBG, fasting blood glucose; 2hPG, 2-h plasma glucose; HOMA%β, homeostasis model assessment of β-cell function; HOMA%S, homeostasis model assessment of insulin sensitivity; HDL-C, high-density lipoprotein cholesterol; LDL-C, low-density lipoprotein cholesterol; HbA1c, glycated hemoglobin; TG, triglycerides; TC, total cholesterol.

Bold P values indicate a significant difference.

**Table 5:** Multivariable linear regression of FINDRISC .

Variable	*b*	*S_b_*	*b’*	*t*	*p*
Constants	-20.439	5.201	–	-3.930	<0.0001
Height	0.008	0.031	0.015	0.261	0.794
Weight	0.056	0.035	0.146	1.622	0.105
Systolic blood pressure	0.010	0.012	0.043	0.807	0.420
Diastolic blood pressure	0.010	0.020	0.025	0.488	0.626
FBG	0.423	0.273	0.074	1.549	0.122
**2hPG**	**0.302**	**0.083**	**0.160**	**3.620**	**<0.0001**
HbA1c	0.255	0.329	0.036	0.777	0.438
Fasting serum insulin	0.003	0.017	0.007	0.181	0.856
HOMA%β	0.000415	0.004	0.008	0.097	0.923
HOMA%S	0.000375	0.001	0.020	0.395	0.693
HOMA-IR	0.066	0.281	0.018	0.236	0.813
TC	0.440	0.462	0.093	0.954	0.341
TG	0.000480	0.157	0.000159	-0.003	0.998
HDL-C	-0.425	0.742	-0.026	-0.574	0.567
LDL-C	-0.362	0.548	-0.056	-0.660	0.510
Dependent variable: FINDRISC score; R^2^ = 0.527; F = 27.287, p < 0.0001					

FBG, fasting blood glucose; 2hPG, 2-h plasma glucose; HOMA%β, homeostasis model assessment of β-cell function; HOMA%S, homeostasis model assessment of insulin sensitivity; HDL-C, high-density lipoprotein cholesterol; LDL-C, low-density lipoprotein cholesterol; HbA1c, glycated hemoglobin; TG, triglycerides; TC, total cholesterol.

Bold text indicates a significant difference.

### Diagnostic Accuracy for Undiagnosed T2DM

**Figure 1:**
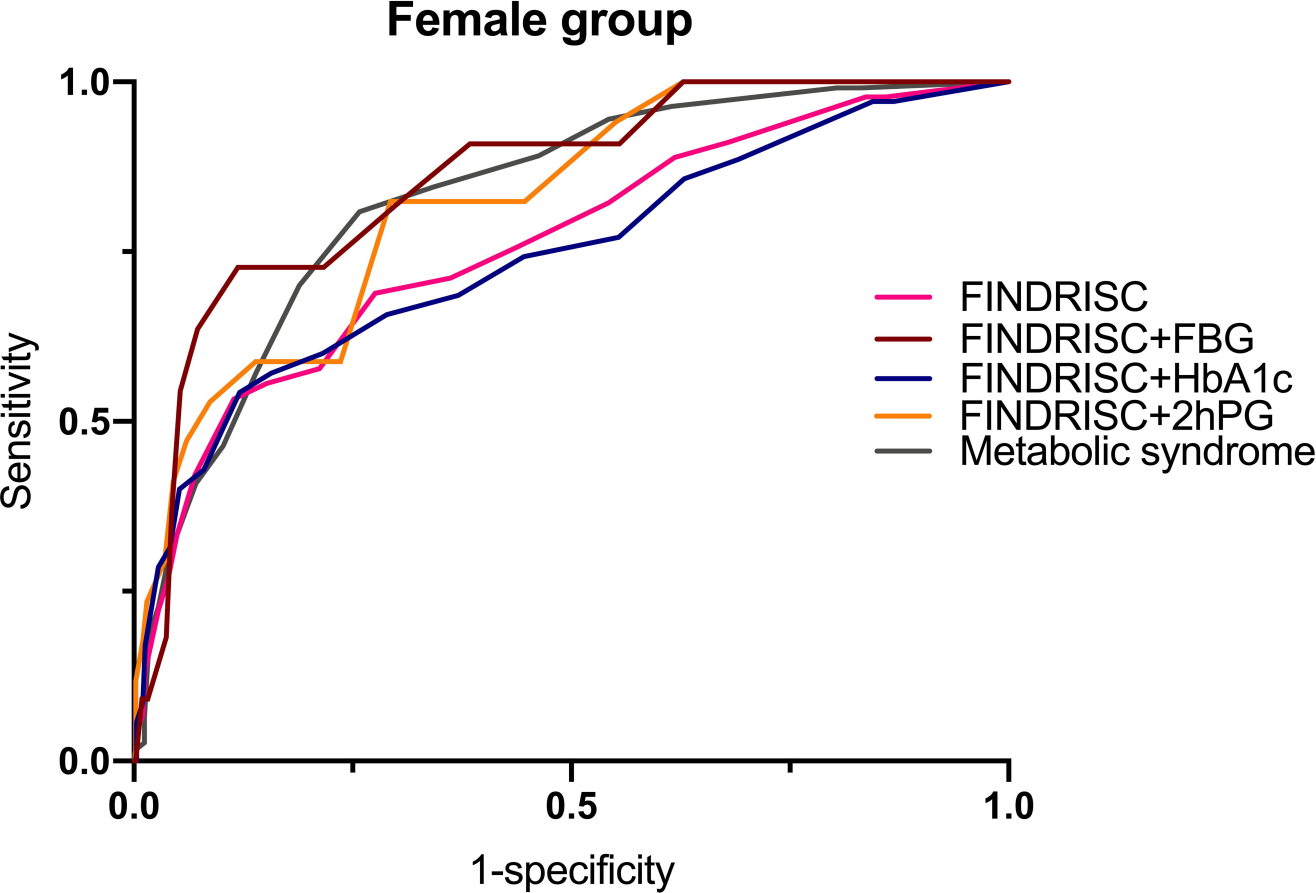
FINDRISC area under ROC curve (AUC) for identifying T2DM in female populations.

**Figure 2:**
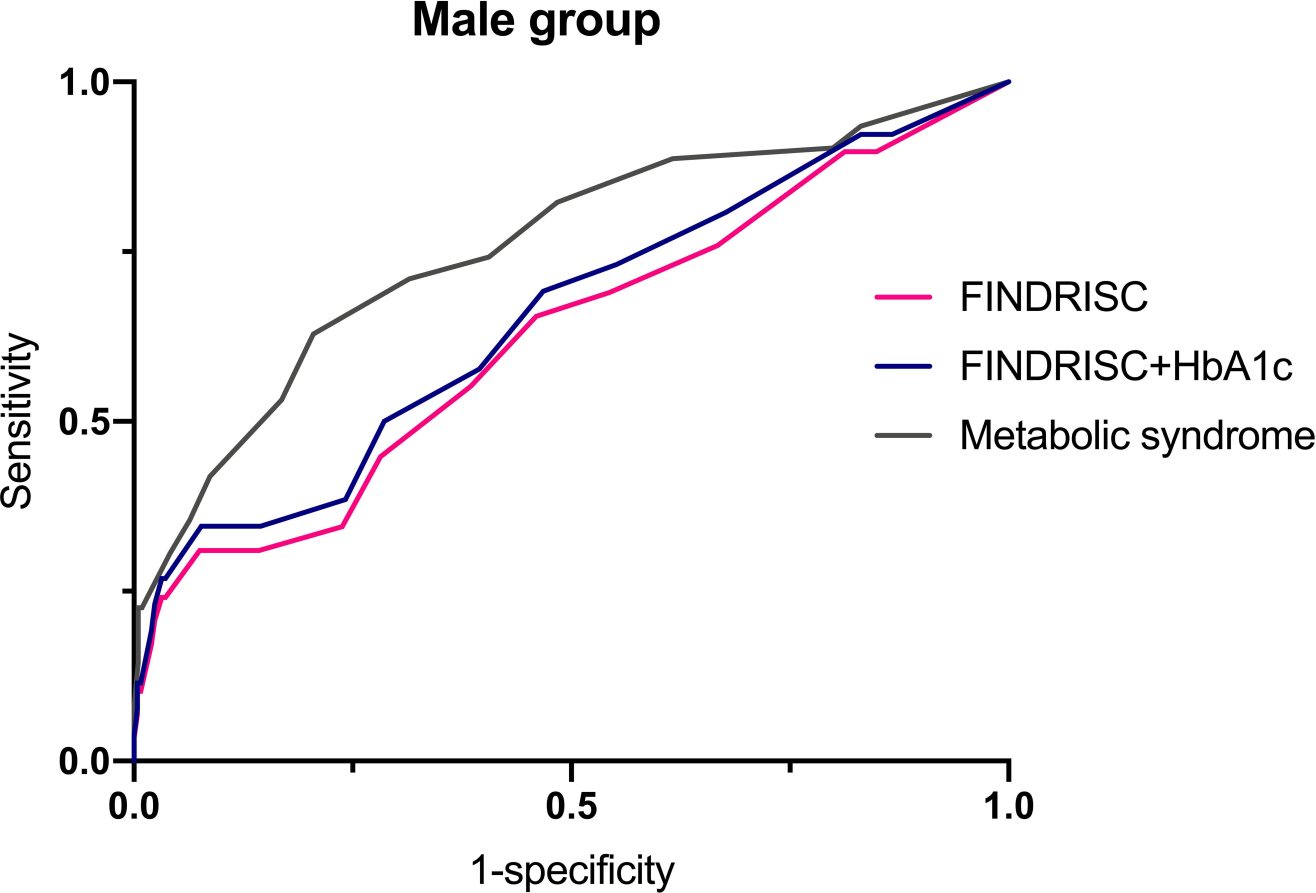
FINDRISC area under ROC curve (AUC) for identifying T2DM in male populations.

**Figure 3:**
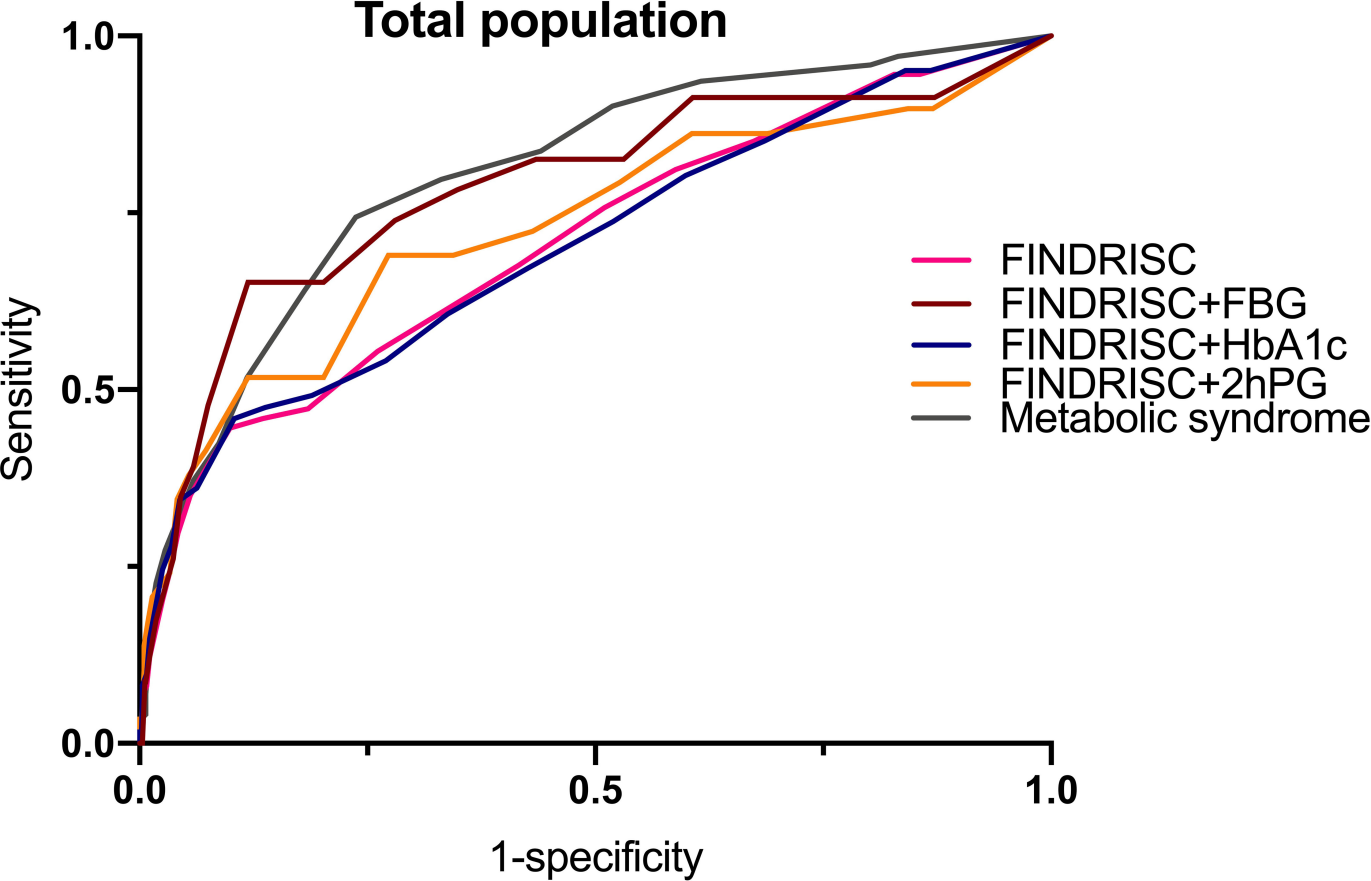
FINDRISC area under ROC curve (AUC) for identifying T2DM in total populations.

**Table 6** shows the cut-off points of the metabolic syndrome and FINDRISC for screening of undiagnosed T2DM using 2hPG, FBG, and HbA1c as the diagnostic criteria, separately for the overall, female, and male populations. The highest AUC-ROC value was observed for the FBG criteria in the FINDRISC (AUC-ROC = 0.858, 95% CI 0.753–0.964) among the female group, followed by the female group with the metabolic syndrome (AUC-ROC = 0.830, 95% CI 0.788–0.872), and using FINDRISC with 2hPG among the female group (AUC-ROC = 0.823, 95% CI 0.730–0.916) (**Figure 1**). FINDRISC and the metabolic syndrome appeared to have better performance in the female group than in the male group (**Figure 2**). The AUC-ROC of the FINDRISC to identify subjects with T2DM among the total population was 0.708 (95% CI 0.639–0.776); its sensitivity was 44.6% and its specificity was 90.1%, with 11 as the cut-off point. When adding FBG or 2hPG to the FINDRISC, the AUC-ROC among the total population significantly increased to 0.785 (95% CI 0.671–0.899) and 0.731 (95% CI 0.619–0.843), respectively, while the AUC-ROC among the female group increased to 0.858 (95% CI 0.753–0.964) and 0.823 (95% CI 0.730–0.916), respectively (p < 0.001) (**Figure 3**). The AUC-ROC of the metabolic syndrome to identify subjects with T2DM among the total and female population was 0.805 (95% CI 0.767–0.844) and 0.830 (95% CI 0.788–0.872), respectively, with seven as the cut-off point.

**Table 6:** FINDRISC area under ROC curve (AUC) for identifying T2DM.

	AUC (95% CI)	Sensitivity	Specificity	Youden’s index	Cut-off	p
FINDRISC (both sexes)	0.708 (0.639–0.776)	44.6%	90.1%	0.347	11	<0.001
FINDRISC (male)	0.625 (0.508–0.742)	31.0%	92.5%	0.235	11	0.028
FINDRISC (female)	0.761 (0.683–0.840)	53.3%	88.6%	0.420	11	<0.001
FBG (both sexes)	0.785 (0.671–0.899)	65.2%	88.1%	0.533	11	<0.001
FBG (male)	0.482 (0.232–0.733)					0.881
FBG (female)	0.858 (0.753–0.964)	72.7%	88.1%	0.609	11	<0.001
HbA1c (both sexes)	0.704 (0.627–0.780)	45.9%	89.6%	0.355	11	<0.001
HbA1c (male)	0.653 (0.532–0.773)	34.6%	92.3%	0.270	11	0.01
HbA1c (female)	0.745 (0.649–0.841)	54.3%	87.9%	0.422	11	<0.001
2hPG (both sexes)	0.731 (0.619–0.843)	69.0%	72.7%	0.416	8	<0.001
2hPG (male)	0.597 (0.380–0.813)					0.258
2hPG (female)	0.823 (0.730–0.916)	82.4%	70.7%	0.531	8	<0.001
MS (both sexes)	0.805 (0.767–0.844)	74.4%	76.3%	0.508	7	<0.001
MS (male)	0.757 (0.683–0.830)	62.9%	79.5%	0.424	7	<0.001
MS (female)	0.830 (0.788–0.872)	80.9%	74.2%	0.551	7	<0.001

FBG, fasting blood glucose; 2hPG, 2-h plasma glucose; HbA1c, glycated hemoglobin; MS, the metabolic syndrome.

While using the FINDRISC model, adding FBG resulted in the highest AUC-ROC value (0.785) among the total population, compared to the addition of HbA1c (0.704) and 2hPG (0.731). Although the addition of FBG or 2hPG increased the AUC-ROC value of FINSDRISC for predicting T2DM, the discriminatory accuracy was lackluster for the metabolic syndrome. A sensitivity analysis of the influence of different threshold values was performed (**Tables S1**–**S15**). When adding FBG or HbA1c, the optimal cut-off point according to the Youden’s index was 11, while adding 2hPG to the FINDRISC model led its cut-off point to decrease to eight. In contrast, the optimal cut-off point for the metabolic syndrome was seven, with a sensitivity and specificity of 74.4% and 76.3%, respectively. For female and male subjects, the optimal cut-off was the same as that for the overall population.

## Discussion

The metabolic syndrome is a cluster of insulin resistance and disturbed glucose metabolism, overweight and abdominal fat distribution, dyslipidemia, and high blood pressure, and commonly occurs in the majority of patients with prediabetes or T2DM (12). Approximately 25% of adults worldwide are estimated to have the metabolic syndrome (22). In addition, patients with the metabolic syndromes have a 5-fold higher risk of developing T2DM than those with the normal metabolic syndrome (13). A study (23) reported that the FINDRISC assessment model exhibited a good ability to predict the metabolic syndrome in a cohort of first-degree relatives of T2DM patients, with an AUC value of 65%. In this study, the prevalence of the metabolic syndrome increased with increasing FINDRISCs. Several cross-sectional studies have assessed the FINDRISC model as a screening tool for the metabolic syndrome. The lipid metabolic indicators (blood pressure, FBG, TC, TG, HDL-C, and LDL-C) were found to be significantly related to the FINDRISC model, which are common risk factors for T2DM (24). This finding indicated that the FINDRISC model could be used for undiagnosed T2DM screening in this cohort. This in turn revealed that a non-invasive screening tool combined with a relatively inexpensive and feasible biochemical marker was a better tool to identify subjects with T2DM, as opposed to a cluster of clinical and biochemical markers for diagnosing the metabolic syndrome (24).

In 2009, Chien (25) used a Cox regression analysis to establish a risk scoring model for T2DM, namely the Taiwan model of China. A total of 2960 subjects aged over 35 years with a 10-year follow-up were selected for this cohort study. Risk factors in this invasive model included TG, HDL-C, FBG, and white blood cell levels. The AUC-ROC value for this model was 0.70. In 2012, a Chinese population (26) was used to test several domestic and foreign T2DM evaluation models, including the FINDRISC and Framingham models. It was found that the Taiwan model of China had the highest predictive efficiency among all models, with AUC-ROC values of 0.75 among all models; these were similar to the AUC-ROC values (0.704 to 0.785) found using the FINDRISC model in this study.

Several diabetes risk-score models have been developed to predict the risk of T2DM. These models can be used in clinical practice to identify people at high risk of T2DM, and to guide clinical treatment (17). However, it is still not clear whether these models can be applied in local populations. The incidence and risk factors of T2DM in a population determine the suitability of a risk score. Some models developed in a particular population always do not perform well in other populations. There have been extensive studies on risk scores that have been developed specifically in Chinese settings. Mao (27) evaluated the performance of The New Chinese Diabetes Risk Score (NCDRS) model in detecting undiagnosed diabetes and prediabetes among 7,675 community residents in Jiangsu Province. The results showed that the participants with undiagnosed diabetes reported the highest NCDRS value, and the best cut-off points of NCDRS for detecting undiagnosed diabetes and prediabetes were 27. Their results indicated the excellent performance of NCDRS in screening undiagnosed diabetes in Jiangsu Province, and further provide evidence for using NCDRS in detecting prediabetes. Zhang (28) developed and validated a prediction model for T2DM (Chinese risk model) in a cohort of rural adult Chinese population. With the validation dataset, the performance of this model was superior to the FINDRISC and the other models of T2DM risk, which was widely applicable for predicting 6-year risk of T2DM in a rural adult Chinese population.

The FINDRISC was originally developed in a prospective cohort to identify individuals at high risk of developing T2DM (29). Previous studies have analyzed the performance of FINDRISCs for the detection of undiagnosed T2DM (30). The FINDRISC model has been verified in several Western populations for T2DM screening (31–34). These results showed that the optimal cut-off points ranged widely from 9 (29) to 15 (33). To date, only four studies have evaluated the use of FINDRISC in Asian populations, including populations in the Philippines, Malaysia, Mongolia, and India (35–38). All of these results suggest that the FINDRISC has a good performance for T2DM screening in the Asian population. However, there was no publication, if any, which focused on the validation of the FINDRISC for the screening of T2DM in a Chinese population.

It has been reported that the performance of a diabetes risk score can be improved by adding biochemical markers (39). The HbA1c test is a more convenient screening method than FBG and 2hPG, which does not require fasting, and may not be influenced by day-to-day variations (40). HbA1c was used to monitor glycemic control in diabetic patients as it can reflect the average blood glucose levels over 2–3 months (41). However, some studies have shown that there is poor concordance between HbA1c and FBG or 2hPG during an OGTT, which are the most widely accepted glucose-based methods for diagnosing T2DM. Several studies (40, 42) employed the WHO diabetes diagnostic criteria or only FBG values in the FINDRISC assessment model among other populations. With the introduction of HbA1c as a diagnostic criterion by the ADA guidelines in 2009, studies evaluating the performance of the FINDRISC model by using HbA1c as the gold standard showed good results, with AUC-ROC values ranging from 0.72 to 0.81 (43–45). A previous study (45) used FBG, 2hPG, or HbA1c as the diagnostic criteria in their FINDRISC assessment model. The results showed a higher sensitivity (75% in men *vs*. 72% in women) and AUC-ROC value (0.75) than other studies that used 2hPG and/or FBG criteria. In contrast, two studies (15, 29) reported a higher AUC-ROC value than Zhang’s study, which used 2hPG and/or FBG as the diagnostic criteria. Similarly, Costa (46) conducted a study to detect undiagnosed T2DM using FBG, 2hPG, and HbA1c alone instead of using combinations of these criteria. Their results suggested that 2hPG and FBG had better discriminatory power than HbA1c, which is in agreement with the observations in this study. In this study, FBG, 2hPG, and HbA1c levels were used as diagnostic criteria. Based on FBG or HbA1c criteria, 11 was the optimal cut-off point for identifying individuals with undiagnosed T2DM, while the optimal cut-off point was eight based on 2hPG criteria. Among these criteria, FBG displayed the best performance in screening for undiagnosed T2DM in the overall population, with an AUC-ROC value of 0.785, followed by 2hPG and HbA1c criteria, with AUC-ROC values of 0.731 and 0.704, respectively. The cut-off points using FBG or HbA1c were higher than those reported in European studies (15, 31, 34, 47). The studies used a value ≥ 9 as the best cut-off point without incorporating the peaks of the ROC curve, where the sum of the sensitivity and specificity is at maximum. In addition, all these diagnostic criteria performed better at screening for undiagnosed T2DM among the female population than for the overall population. However, FINDRISCs with 2hPG and FBG failed to screen for undiagnosed T2DM among the male population.

The discriminatory accuracy in identifying subjects with diabetes of the original FINDRISC questionnaire was similar to that of the metabolic syndrome; however, the FINDRISC model is much easier to perform, as it did not require invasive testing. Despite the different performance when using different diagnostic criteria, the findings in this study indicated that the FINDRISC could be used as an initial screening tool to help clinicians identify patients at high risk of T2DM. In addition, FINDRISC with FBG and a cut-off point of 11 had a higher discriminative power for screening T2DM compared to 2hPG and HbA1c. To the best of our knowledge, this study is the first to assess the applicability of the FINDRISC model in a Chinese population. Even though the metabolic syndrome showed a better performance than using the FINDRISC screening model, adding FBG or 2hPG significantly increased the discriminative power of the FINDRISC. However, this study had several limitations. First, in this project, the sample size was relatively small, which was calculated based primarily on outpatients with risk factors who visited the hospital during the period from July 2019 to March 2020. The reason why the morbidity of this population is lower than that of Chinese population should be caused by insufficient sample size and population selection, which needs to be further proved by subsequent experiments. Further studies with larger sample sizes are needed to include conclusive data for clinical practice. Second, as participants were recruited from the districts in the metropolitan area of Shanghai, the results may not be applicable to the rest of the population of China.

## Conclusion

In this study, we tried first to measure the FINDRISC model’s performance in predicting current glucose disorders, and then to compare the results based on different sets of diagnostic criteria, which is one of the strengths of this study. According to our findings, the simple scoring model is less sensitive to the prediction of T2DM. Only when FBG or 2hPG was introduced, the prediction sensitivity of the model increased. It seems that the application of FINDRISC to predict T2DM might be less important. However, it is worth noting that including FPG, 2hPG, and HbA1c levels into the model introduces a bias, as these parameters are commonly used for the diagnosis of T2DM itself, which could falsely increase the discriminative power of this risk model. Nevertheless, a non-invasive screening tool with a relatively inexpensive and feasible biochemical marker is a better tool to identify subjects with diabetes than a cluster of clinical and biochemical markers. Hence, our findings may prompt the application of T2DM in a two-step model among the Chinese population.

## Data Availability Statement

The original contributions presented in the study are included in the article/**Supplementary Material**. Further inquiries can be directed to the corresponding authors.

## Ethics Statement

The studies involving human participants were reviewed and approved by Ethics Committee of Shuguang Hospital affiliated to Shanghai University of Traditional Chinese Medicine. The patients/participants provided their written informed consent to participate in this study.

## Author Contributions

ZY and HL substantially contributed to the general idea and design of the study. SYJ completed the data analysis and the manuscript. QGC, XH, YHL, and MJC assisted in case collection and clinical data recording. All authors contributed to the article and approved the submitted version.

## Funding

This study was supported by the Shanghai Municipal Key Clinical Specialty (No. shslczdzk05401) and the Shanghai Key Laboratory of Traditional Chinese Clinical Medicine (No. 14DZ2273200) projects.

## Conflict of Interest

The authors declare that the research was conducted in the absence of any commercial or financial relationships that could be construed as a potential conflict of interest.

## Publisher’s Note

All claims expressed in this article are solely those of the authors and do not necessarily represent those of their affiliated organizations, or those of the publisher, the editors and the reviewers. Any product that may be evaluated in this article, or claim that may be made by its manufacturer, is not guaranteed or endorsed by the publisher.

## References

[1] Bommer C Sagalova V Heesemann E Manne-Goehler J Atun R Bärnighausen T. Global Economic Burden of Diabetes in Adults: Projections From 2015 to 2030. Diabetes Care (2018) 41(5):963–70. doi: 10.2337/dc17-1962 29475843

[2] IDF. IDF DIABETES ATLAS, Ninth edition (2019). Available at: https://www.diabetesatlas.org/upload/resources/material/20200302_133351_IDFATLAS9e-final-web.pdf(2019).

[3] Li Y Teng D Shi X Qin G Qin Y Quan H. Prevalence of Diabetes Recorded in Mainland China Using 2018 Diagnostic Criteria From the American Diabetes Association: National Cross Sectional Study. BMJ (2020) 369:m997. doi: 10.1136/bmj.m997 32345662 PMC7186854

[4] Galaviz KI Narayan KMV Lobelo F Weber MB. Lifestyle and the Prevention of Type 2 Diabetes: A Status Report. Am J Lifestyle Med (2015) 12(1):4–20. doi: 10.1177/1559827615619159 30202378 PMC6125024

[5] Diabetes Prevention Program Research Group Knowler WC Fowler SE Hamman RF Christophi CA Hoffman HJ. 10-Year Follow-Up of Diabetes Incidence and Weight Loss in the Diabetes Prevention Program Outcomes Study. Lancet (2009) 374(9702):1677–86. doi: 10.1016/S0140-6736(09)61457-4 PMC3135022 19878986

[6] Haw JS Galaviz KI Straus AN Kowalski AJ Magee MJ Weber MB. Long-Term Sustainability of Diabetes Prevention Approaches: A Systematic Review and Meta-Analysis of Randomized Clinical Trials. JAMA Intern Med (2017) 177(12):1808–17. doi: 10.1001/jamainternmed.2017.6040 PMC5820728 29114778

[7] Li G Zhang P Wang J Gregg EW Yang W Gong Q. The Long-Term Effect of Lifestyle Interventions to Prevent Diabetes in the China Da Qing Diabetes Prevention Study: A 20-Year Follow-Up Study. Lancet (2008) 371(9626):1783–9. doi: 10.1016/S0140-6736(08)60766-7 18502303

[8] Tuomilehto J Lindström J Eriksson JG Valle TT Hämäläinen H Ilanne-Parikka P. Prevention of Type 2 Diabetes Mellitus by Changes in Lifestyle Among Subjects With Impaired Glucose Tolerance. N Engl J Med (2001) 344(18):1343–50. doi: 10.1056/nejm200105033441801 11333990

[9] Ong SE Koh JJK Toh S-AES Chia KS Balabanova D McKee M. Assessing the Influence of Health Systems on Type 2 Diabetes Mellitus Awareness, Treatment, Adherence, and Control: A Systematic Review. PloS One (2018) 13(3):e0195086-e. doi: 10.1371/journal.pone.0195086 29596495 PMC5875848

[10] Ma M Liu H Yu J He S Li P Ma C. Triglyceride is Independently Correlated With Insulin Resistance and Islet Beta Cell Function: A Study in Population With Different Glucose and Lipid Metabolism States. Lipids Health Dis (2020) 19(1):121. doi: 10.1186/s12944-020-01303-w 32487177 PMC7268278

[11] Song Y Manson JE Tinker L Howard BV Kuller LH Nathan L. Insulin Sensitivity and Insulin Secretion Determined by Homeostasis Model Assessment and Risk of Diabetes in a Multiethnic Cohort of Women: The Women's Health Initiative Observational Study. Diabetes Care (2007) 30(7):1747–52. doi: 10.2337/dc07-0358 PMC1952235 17468352

[12] Bansal N. Prediabetes Diagnosis and Treatment: A Review. World J Diabetes (2015) 6(2):296–303. doi: 10.4239/wjd.v6.i2.296 25789110 PMC4360422

[13] Tabák AG Herder C Rathmann W Brunner EJ Kivimäki M. Prediabetes: A High-Risk State for Diabetes Development. Lancet (2012) 379(9833):2279–90. doi: 10.1016/S0140-6736(12)60283-9 PMC3891203 22683128

[14] Expert Panel on Detection, Evaluation, and Treatment of High Blood Cholesterol in Adults. Executive Summary of the Third Report of the National Cholesterol Education Program (NCEP) Expert Panel on Detection, Evaluation, and Treatment of High Blood Cholesterol in Adults (Adult Treatment Panel III). JAMA (2001) 285(19):2486–97. doi: 10.1001/jama.285.19.2486 11368702

[15] Li J Bergmann A Reimann M Bornstein SR Schwarz PE. A More Simplified Finnish Diabetes Risk Score for Opportunistic Screening of Undiagnosed Type 2 Diabetes in a German Population With a Family History of the Metabolic Syndrome. Horm Metab Res (2009) 41(2):98–103. doi: 10.1055/s-0028-1087191 18975253

[16] Wu Y Ding Y Tanaka Y Zhang W. Risk Factors Contributing to Type 2 Diabetes and Recent Advances in the Treatment and Prevention. Int J Med Sci (2014) 11(11):1185–200. doi: 10.7150/ijms.10001 PMC4166864 25249787

[17] Buijsse B Simmons RK Griffin SJ Schulze MB. Risk Assessment Tools for Identifying Individuals at Risk of Developing Type 2 Diabetes. Epidemiol Rev (2011) 33(1):46–62. doi: 10.1093/epirev/mxq019 21622851 PMC3132807

[18] Kundu S Kers JG Janssens ACJW. Constructing Hypothetical Risk Data From the Area Under the ROC Curve: Modelling Distributions of Polygenic Risk. PloS One (2016) 11(3):e0152359. doi: 10.1371/journal.pone.0152359 27023073 PMC4811433

[19] WHO. Definition and Diagnosis of Diabetes Mellitus and Intermediate Hyperglycaemia . Available at: https://www.who.int/diabetes/publications/Definition%20and%20diagnosis%20of%20diabetes_new.pdf(2013).

[20] ADA. Standards of Medical Care in Diabetes—2013. Diabetes Care (2013) 36(Supplement 1):S11–66. doi: 10.2337/dc13-S011 PMC3537269 23264422

[21] WHO. Appropriate Body-Mass Index for Asian Populations and its Implications for Policy and Intervention Strategies. Lancet (2004) 363(9403):157–63. doi: 10.1016/S0140-6736(03)15268-3 14726171

[22] Saklayen MG. The Global Epidemic of the Metabolic Syndrome. Curr Hypertens Rep (2018) 20(2):12–. doi: 10.1007/s11906-018-0812-z PMC5866840 29480368

[23] Janghorbani M Adineh H Amini M. Evaluation of the Finnish Diabetes Risk Score (FINDRISC) as a Screening Tool for the Metabolic Syndrome. Rev Diabetes Stud (2013) 10(4):283–92. doi: 10.1900/RDS.2013.10.283 PMC4160014 24841881

[24] Meijnikman AS De Block CEM Verrijken A Mertens I Van Gaal LF. Predicting Type 2 Diabetes Mellitus: A Comparison Between the FINDRISC Score and the Metabolic Syndrome. Diabetol Metab Syndr (2018) 10:12–. doi: 10.1186/s13098-018-0310-0 PMC5831861 29507612

[25] Chien K Cai T Hsu H Su T Chang W Chen M. A Prediction Model for Type 2 Diabetes Risk Among Chinese People. Diabetologia (2008) 52(3):443. doi: 10.1007/s00125-008-1232-4 19057891

[26] He S Chen X Cui K Peng Y Liu K Lv Z. Validity Evaluation of Recently Published Diabetes Risk Scoring Models in a General Chinese Population. Diabetes Res Clin Pract (2012) 95(2):291–8. doi: 10.1016/j.diabres.2011.10.039 22129653

[27] Mao T Chen J Guo H Qu C He C Xu X. The Efficacy of New Chinese Diabetes Risk Score in Screening Undiagnosed Type 2 Diabetes and Prediabetes: A Community-Based Cross-Sectional Study in Eastern China. J Diabetes Res (2020) 2020:7463082. doi: 10.1155/2020/7463082 32405505 PMC7210548

[28] Zhang M Zhang H Wang C Ren Y Wang B Zhang L. Development and Validation of a Risk-Score Model for Type 2 Diabetes: A Cohort Study of a Rural Adult Chinese Population. PloS One (2016) 11(4):e0152054-e. doi: 10.1371/journal.pone.0152054 27070555 PMC4829145

[29] Lindström J Tuomilehto J. The Diabetes Risk Score: A Practical Tool to Predict Type 2 Diabetes Risk. Diabetes Care (2003) 26(3):725–31. doi: 10.2337/diacare.26.3.725 12610029

[30] Salinero-Fort M de Burgos-Lunar C Mostaza Prieto J Lahoz Rallo C Abánades-Herranz JC Gómez-Campelo P. Validating Prediction Scales of Type 2 Diabetes Mellitus in Spain: The SPREDIA-2 Population-Based Prospective Cohort Study Protocol. BMJ Open (2015) 5(7):e007195. doi: 10.1136/bmjopen-2014-007195 PMC4521512 26220868

[31] Franciosi M De Berardis G Rossi MCE Sacco M Belfiglio M Pellegrini F. Use of the Diabetes Risk Score for Opportunistic Screening of Undiagnosed Diabetes and Impaired Glucose Tolerance: The IGLOO (Impaired Glucose Tolerance and Long-Term Outcomes Observational) Study. Diabetes Care (2005) 28(5):1187–94. doi: 10.2337/diacare.28.5.1187 15855587

[32] Hellgren MI Petzold M Björkelund C Wedel H Jansson P-A Lindblad U. Feasibility of the FINDRISC Questionnaire to Identify Individuals With Impaired Glucose Tolerance in Swedish Primary Care. A Cross-Sectional Population-Based Study. Diabetic Med (2012) 29(12):1501–5. doi: 10.1111/j.1464-5491.2012.03664.x 22443428

[33] Makrilakis K Liatis S Grammatikou S Perrea D Stathi C Tsiligros P. Validation of the Finnish Diabetes Risk Score (FINDRISC) Questionnaire for Screening for Undiagnosed Type 2 Diabetes, Dysglycaemia and the Metabolic Syndrome in Greece. Diabetes Metab (2011) 37(2):144–51. doi: 10.1016/j.diabet.2010.09.006 21144787

[34] Soriguer F Valdés S Tapia MJ Esteva I Ruiz de Adana MS Almaraz MC. Validación Del FINDRISC (FINnish Diabetes Risk SCore) Para La Predicción Del Riesgo De Diabetes Tipo 2 En Una Población Del Sur De España. Estudio Pizarra. Med Clín (2012) 138(9):371–6. doi: 10.1016/j.medcli.2011.05.025 21939990

[35] Dugee O Janchiv O Jousilahti P Sakhiya A Palam E Nuorti JP. Adapting Existing Diabetes Risk Scores for an Asian Population: A Risk Score for Detecting Undiagnosed Diabetes in the Mongolian Population. BMC Public Health (2015) 15(1):938. doi: 10.1186/s12889-015-2298-9 26395572 PMC4578253

[36] Ku GMV Kegels G. The Performance of the Finnish Diabetes Risk Score, a Modified Finnish Diabetes Risk Score and a Simplified Finnish Diabetes Risk Score in Community-Based Cross-Sectional Screening of Undiagnosed Type 2 Diabetes in the Philippines. Prim Care Diabetes (2013) 7(4):249–59. doi: 10.1016/j.pcd.2013.07.004 23953706

[37] Lim HM Chia YC Koay ZL. Performance of the Finnish Diabetes Risk Score (FINDRISC) and Modified Asian FINDRISC (ModAsian FINDRISC) for Screening of Undiagnosed Type 2 Diabetes Mellitus and Dysglycaemia in Primary Care. Prim Care Diabetes (2020) 14(5):494–500. doi: 10.1016/j.pcd.2020.02.008 32156516

[38] Pawar SD Naik JD Prabhu P Jatti GM Jadhav SB Radhe BK. Comparative Evaluation of Indian Diabetes Risk Score and Finnish Diabetes Risk Score for Predicting Risk of Diabetes Mellitus Type II: A Teaching Hospital-Based Survey in Maharashtra. J Family Med Prim Care (2017) 6(1):120–5. doi: 10.4103/2249-4863.214957 PMC5629875 29026763

[39] Saaristo T Peltonen M Lindström J Saarikoski L Sundvall J Eriksson JG. Cross-Sectional Evaluation of the Finnish Diabetes Risk Score: A Tool to Identify Undetected Type 2 Diabetes, Abnormal Glucose Tolerance and Metabolic Syndrome. Diabetes Vasc Dis Res (2005) 2(2):67–72. doi: 10.3132/dvdr.2005.011 16305061

[40] Getaneh A Andres R Brillon DJ Findley SE. Hemoglobin A1C Criterion for Diabetes Diagnosis Among Hispanic and Non-Hispanic Populations. Endocr Pract (2011) 17(2):210–7. doi: 10.4158/EP10119.OR 20841311

[41] Sherwani SI Khan HA Ekhzaimy A Masood A Sakharkar MK. Significance of HbA1c Test in Diagnosis and Prognosis of Diabetic Patients. Biomark Insights (2016) 11:95–104. doi: 10.4137/BMI.S38440 27398023 PMC4933534

[42] Bennett CM Guo M Dharmage SC. HbA1c as a Screening Tool for Detection of Type 2 Diabetes: A Systematic Review. Diabetic Med (2007) 24(4):333–43. doi: 10.1111/j.1464-5491.2007.02106.x 17367307

[43] Gomez-Arbelaez D Alvarado-Jurado L Ayala-Castillo M Forero-Naranjo L Camacho PA Lopez-Jaramillo P. Evaluation of the Finnish Diabetes Risk Score to Predict Type 2 Diabetes Mellitus in a Colombian Population: A Longitudinal Observational Study. World J Diabetes (2015) 6(17):1337–44. doi: 10.4239/wjd.v6.i17.1337 PMC4673387 26675051

[44] Salinero-Fort MA Burgos-Lunar C Lahoz C Mostaza JM Abánades-Herranz JC Laguna-Cuesta F. Performance of the Finnish Diabetes Risk Score and a Simplified Finnish Diabetes Risk Score in a Community-Based, Cross-Sectional Programme for Screening of Undiagnosed Type 2 Diabetes Mellitus and Dysglycaemia in Madrid, Spain: The SPREDIA-2 Study. PloS One (2016) 11(7):e0158489. doi: 10.1371/journal.pone.0158489 27441722 PMC4956208

[45] Zhang L Zhang Z Zhang Y Hu G Chen L. Evaluation of Finnish Diabetes Risk Score in Screening Undiagnosed Diabetes and Prediabetes Among U.S. Adults by Gender and Race: NHANES 1999-2010. PloS One (2014) 9(5):e97865. doi: 10.1371/journal.pone.0097865 24852786 PMC4031122

[46] Costa B Barrio F Piñol JL Cabré JJ Mundet X Sagarra R. Shifting From Glucose Diagnosis to the New HbA1c Diagnosis Reduces the Capability of the Finnish Diabetes Risk Score (FINDRISC) to Screen for Glucose Abnormalities Within a Real-Life Primary Healthcare Preventive Strategy. BMC Med (2013) 11:45–. doi: 10.1186/1741-7015-11-45 PMC3621796 23438147

[47] Witte DR Shipley MJ Marmot MG Brunner EJ. Performance of Existing Risk Scores in Screening for Undiagnosed Diabetes: An External Validation Study. Diabetic Med (2010) 27(1):46–53. doi: 10.1111/j.1464-5491.2009.02891.x 20121888

