# Supplementary material for: Comparison of the Finnish Diabetes Risk Score Model With the Metabolic Syndrome in a Shanghai Population
Source: Front Endocrinol (Lausanne). 2022 Feb 22;13:725314. doi: 10.3389/fendo.2022.725314 (PMC8902815; doi:10.3389/fendo.2022.725314)
Supplement: Supplementary file 1 [file DataSheet_1.docx]

Table S1. Use fasting blood glucose as the diagnostic criterion in total population

| Threshold values | Youden' index | Sensitivity (%) | Specificity (%) |
| --- | --- | --- | --- |
| -1 | 0.000 | 100.0 | 0.00 |
| 1 | 0.041 | 91.3 | 12.8 |
| 2 | 0.067 | 91.3 | 15.4 |
| 3 | 0.221 | 91.3 | 30.8 |
| 4 | 0.306 | 91.3 | 39.3 |
| 5 | 0.295 | 82.6 | 46.9 |
| 6 | 0.391 | 82.6 | 56.5 |
| 7 | 0.434 | 78.3 | 65.1 |
| 8 | 0.459 | 73.9 | 72.0 |
| 9 | 0.450 | 65.2 | 79.8 |
| 10 | 0.498 | 65.2 | 84.6 |
| 11 | 0.533 | 65.2 | 88.1 |
| 12 | 0.403 | 47.8 | 92.5 |
| 13 | 0.333 | 39.1 | 94.1 |
| 14 | 0.302 | 34.8 | 95.5 |
| 15 | 0.224 | 26.1 | 96.3 |
| 16 | 0.156 | 17.4 | 98.2 |
| 17 | 0.119 | 13.0 | 98.8 |
| 18 | 0.080 | 08.7 | 99.3 |
| 19 | 0.081 | 08.7 | 99.4 |
| 20 | -0.003 | 0.00 | 99.7 |
| 22 | -0.001 | 0.00 | 99.9 |
| 25 | 0.000 | 0.00 | 100.0 |

Table S2. Use fasting blood glucose as the diagnostic criterion in male group

| Threshold values | Youden' index | Sensitivity (%) | Specificity (%) |
| --- | --- | --- | --- |
| -1 | 0.000 | 100.0 | 0.00 |
| 1 | -0.037 | 83.3 | 12.9 |
| 2 | -0.183 | 66.7 | 15.1 |
| 3 | -0.041 | 66.7 | 29.2 |
| 4 | 0.052 | 66.7 | 38.5 |
| 5 | -0.041 | 50.0 | 45.9 |
| 6 | 0.050 | 50.0 | 55.0 |
| 7 | -0.023 | 33.3 | 64.4 |
| 8 | 0.025 | 33.3 | 69.1 |
| 9 | -0.051 | 16.7 | 78.2 |
| 10 | -0.018 | 16.7 | 81.6 |
| 11 | 0.021 | 16.7 | 85.4 |
| 12 | 0.078 | 16.7 | 91.1 |
| 13 | 0.093 | 16.7 | 92.6 |
| 14 | -0.060 | 0.0 | 94.0 |
| 15 | -0.045 | 0.0 | 95.5 |
| 16 | -0.019 | 0.0 | 98.1 |
| 17 | -0.017 | 0.0 | 98.3 |
| 18 | -0.014 | 0.0 | 98.6 |
| 19 | -0.012 | 0.0 | 98.8 |
| 22 | -0.002 | 0.0 | 99.8 |
| 25 | 0.000 | 0.0 | 100.0 |

Table S3. Use fasting blood glucose as the diagnostic criterion in female group

| Threshold values | Youden' index | Sensitivity (%) | Specificity (%) |
| --- | --- | --- | --- |
| -1 | 0.000 | 100.0 | 0.00 |
| 1 | 0.132 | 100.0 | 13.2 |
| 2 | 0.153 | 100.0 | 15.3 |
| 3 | 0.304 | 100.0 | 30.4 |
| 4 | 0.372 | 100.0 | 37.2 |
| 5 | 0.354 | 90.9 | 44.5 |
| 6 | 0.455 | 90.9 | 54.6 |
| 7 | 0.526 | 90.9 | 61.6 |
| 8 | 0.519 | 81.8 | 70.1 |
| 9 | 0.510 | 72.7 | 78.3 |
| 10 | 0.567 | 72.7 | 84.0 |
| 11 | 0.609 | 72.7 | 88.1 |
| 12 | 0.563 | 63.6 | 92.7 |
| 13 | 0.493 | 54.5 | 94.7 |
| 14 | 0.320 | 36.4 | 95.7 |
| 15 | 0.145 | 18.2 | 96.3 |
| 16 | 0.075 | 9.1 | 98.4 |
| 17 | 0.082 | 9.1 | 99.1 |
| 19 | -0.002 | 0.00 | 99.8 |
| 21 | 0.000 | 0.00 | 100.0 |

Table S4. Use HbA1c as the diagnostic criterion in total population

| Threshold values | Youden' index | Sensitivity (%) | Specificity (%) |
| --- | --- | --- | --- |
| -1 | 0.000 | 100.0 | 0.00 |
| 1 | 0.083 | 95.1 | 13.2 |
| 2 | 0.111 | 95.1 | 16.0 |
| 3 | 0.167 | 85.2 | 31.4 |
| 4 | 0.204 | 80.3 | 40.1 |
| 5 | 0.217 | 73.8 | 48.0 |
| 6 | 0.246 | 67.2 | 57.4 |
| 7 | 0.269 | 60.7 | 66.2 |
| 8 | 0.271 | 54.1 | 73.0 |
| 9 | 0.303 | 49.2 | 81.1 |
| 10 | 0.337 | 47.5 | 86.2 |
| 11 | 0.355 | 45.9 | 89.6 |
| 12 | 0.298 | 36.1 | 93.7 |
| 13 | 0.300 | 34.4 | 95.6 |
| 14 | 0.244 | 27.9 | 96.5 |
| 15 | 0.221 | 24.6 | 97.5 |
| 16 | 0.137 | 14.8 | 98.9 |
| 17 | 0.090 | 9.80 | 99.2 |
| 18 | 0.079 | 8.20 | 99.7 |
| 19 | 0.062 | 6.60 | 99.7 |
| 20 | 0.015 | 1.60 | 99.8 |
| 22 | 0.016 | 1.60 | 100.0 |
| 25 | 0.000 | 0.00 | 100.0 |

Table S5. Use HbA1c as the diagnostic criterion in male group

| Threshold values | Youden' index | Sensitivity (%) | Specificity (%) |
| --- | --- | --- | --- |
| -1 | 0.000 | 100.0 | 0.00 |
| 1 | 0.056 | 92.3 | 13.3 |
| 2 | 0.092 | 92.3 | 16.9 |
| 3 | 0.130 | 80.8 | 32.3 |
| 4 | 0.178 | 73.1 | 44.8 |
| 5 | 0.225 | 69.2 | 53.2 |
| 6 | 0.182 | 57.7 | 60.5 |
| 7 | 0.214 | 50.0 | 71.4 |
| 8 | 0.143 | 38.5 | 75.8 |
| 9 | 0.201 | 34.6 | 85.5 |
| 10 | 0.237 | 34.6 | 89.1 |
| 11 | 0.270 | 34.6 | 92.3 |
| 12 | 0.233 | 26.9 | 96.4 |
| 13 | 0.237 | 26.9 | 96.8 |
| 14 | 0.207 | 23.1 | 97.6 |
| 15 | 0.172 | 19.2 | 98.0 |
| 16 | 0.107 | 11.5 | 99.2 |
| 17 | 0.111 | 11.5 | 99.6 |
| 19 | 0.073 | 7.7 | 99.6 |
| 22 | 0.038 | 3.8 | 100.0 |
| 25 | 0.000 | 0.00 | 100.0 |

Table S6. Use HbA1c as the diagnostic criterion in female group

| Threshold values | Youden' index | Sensitivity (%) | Specificity (%) |
| --- | --- | --- | --- |
| -1 | 0.000 | 100.0 | 0.00 |
| 1 | 0.103 | 97.1 | 13.1 |
| 2 | 0.126 | 97.1 | 15.5 |
| 3 | 0.195 | 88.6 | 30.9 |
| 4 | 0.228 | 85.7 | 37.1 |
| 5 | 0.217 | 77.1 | 44.6 |
| 6 | 0.297 | 74.3 | 55.4 |
| 7 | 0.315 | 68.6 | 62.9 |
| 8 | 0.368 | 65.7 | 71.1 |
| 9 | 0.384 | 60.0 | 78.4 |
| 10 | 0.414 | 57.1 | 84.3 |
| 11 | 0.422 | 54.3 | 87.9 |
| 12 | 0.349 | 42.9 | 92.0 |
| 13 | 0.348 | 40.0 | 94.8 |
| 14 | 0.273 | 31.4 | 95.9 |
| 15 | 0.257 | 28.6 | 97.2 |
| 16 | 0.159 | 17.1 | 98.7 |
| 17 | 0.075 | 8.6 | 99.0 |
| 18 | 0.055 | 5.7 | 99.7 |
| 20 | -0.003 | 0.00 | 99.7 |
| 21 | 0.000 | 0.00 | 100.0 |

Table S7. Use 2hPG as the diagnostic criterion in total population

| Threshold values | Youden' index | Sensitivity (%) | Specificity (%) |
| --- | --- | --- | --- |
| -1 | 0.000 | 100.0 | 0.00 |
| 1 | 0.026 | 89.7 | 13.0 |
| 2 | 0.054 | 89.7 | 15.7 |
| 3 | 0.173 | 86.2 | 31.1 |
| 4 | 0.256 | 86.2 | 39.4 |
| 5 | 0.266 | 79.3 | 47.3 |
| 6 | 0.294 | 72.4 | 56.9 |
| 7 | 0.345 | 69.0 | 65.6 |
| 8 | 0.416 | 69.0 | 72.7 |
| 9 | 0.315 | 51.7 | 79.8 |
| 10 | 0.363 | 51.7 | 84.6 |
| 11 | 0.398 | 51.7 | 88.1 |
| 12 | 0.341 | 41.4 | 92.7 |
| 13 | 0.325 | 37.9 | 94.6 |
| 14 | 0.304 | 34.5 | 95.9 |
| 15 | 0.208 | 24.1 | 96.7 |
| 16 | 0.193 | 20.7 | 98.6 |
| 17 | 0.163 | 17.2 | 99.1 |
| 18 | 0.133 | 13.8 | 99.5 |
| 19 | 0.099 | 10.3 | 99.5 |
| 20 | 0.033 | 3.4 | 99.8 |
| 22 | 0.034 | 3.4 | 100.0 |
| 25 | 0.000 | 0.00 | 100.0 |

Table S8. Use 2hPG as the diagnostic criterion in male group

| Threshold values | Youden' index | Sensitivity (%) | Specificity (%) |
| --- | --- | --- | --- |
| -1 | 0.000 | 100.0 | 0.00 |
| 1 | -0.126 | 75.0 | 12.4 |
| 2 | -0.092 | 75.0 | 15.8 |
| 3 | -0.024 | 66.7 | 30.9 |
| 4 | 0.095 | 66.7 | 42.9 |
| 5 | 0.093 | 58.3 | 51.0 |
| 6 | 0.178 | 58.3 | 59.5 |
| 7 | 0.203 | 50.0 | 70.3 |
| 8 | 0.257 | 50.0 | 75.7 |
| 9 | 0.266 | 41.7 | 84.9 |
| 10 | 0.297 | 41.7 | 88.0 |
| 11 | 0.328 | 41.7 | 91.1 |
| 12 | 0.200 | 25.0 | 95.0 |
| 13 | 0.204 | 25.0 | 95.4 |
| 14 | 0.215 | 25.0 | 96.5 |
| 15 | 0.136 | 16.7 | 96.9 |
| 16 | 0.155 | 16.7 | 98.8 |
| 17 | 0.159 | 16.7 | 99.2 |
| 19 | 0.076 | 8.3 | 99.2 |
| 22 | 0.083 | 8.3 | 100.0 |
| 25 | 0.000 | 0.00 | 100.0 |

Table S9. Use 2hPG as the diagnostic criterion in female group

| Threshold values | Youden' index | Sensitivity (%) | Specificity (%) |
| --- | --- | --- | --- |
| -1 | 0.000 | 100.0 | 0.0 |
| 1 | 0.134 | 100.0 | 13.4 |
| 2 | 0.156 | 100.0 | 15.6 |
| 3 | 0.313 | 100.0 | 31.3 |
| 4 | 0.372 | 100.0 | 37.2 |
| 5 | 0.390 | 94.1 | 44.9 |
| 6 | 0.377 | 82.4 | 55.3 |
| 7 | 0.449 | 82.4 | 62.5 |
| 8 | 0.531 | 82.4 | 70.7 |
| 9 | 0.353 | 58.8 | 76.4 |
| 10 | 0.412 | 58.8 | 82.4 |
| 11 | 0.449 | 58.8 | 86.1 |
| 12 | 0.443 | 52.9 | 91.3 |
| 13 | 0.411 | 47.1 | 94.0 |
| 14 | 0.367 | 41.2 | 95.5 |
| 15 | 0.259 | 29.4 | 96.5 |
| 16 | 0.220 | 23.5 | 98.5 |
| 17 | 0.167 | 17.6 | 99.0 |
| 18 | 0.115 | 11.8 | 99.8 |
| 20 | -0.002 | 0.0 | 99.8 |
| 21 | 0.000 | 0.0 | 100.0 |

Table S10. Threshold values of original FINDRISC in total population

| Threshold values | Youden' index | Sensitivity (%) | Specificity (%) |
| --- | --- | --- | --- |
| -1 | 0.000 | 100.0 | 0.0 |
| 1 | 0.090 | 94.6 | 14.4 |
| 2 | 0.118 | 94.6 | 17.2 |
| 3 | 0.177 | 85.1 | 32.6 |
| 4 | 0.222 | 81.1 | 41.2 |
| 5 | 0.247 | 75.7 | 49.0 |
| 6 | 0.259 | 67.6 | 58.4 |
| 7 | 0.278 | 60.8 | 67.0 |
| 8 | 0.293 | 55.4 | 73.9 |
| 9 | 0.288 | 47.3 | 81.5 |
| 10 | 0.325 | 45.9 | 86.5 |
| 11 | 0.347 | 44.6 | 90.1 |
| 12 | 0.295 | 35.1 | 94.4 |
| 13 | 0.255 | 29.7 | 95.8 |
| 14 | 0.210 | 24.3 | 96.7 |
| 15 | 0.178 | 20.3 | 97.5 |
| 16 | 0.111 | 12.2 | 98.9 |
| 17 | 0.073 | 8.1 | 99.2 |
| 18 | 0.064 | 6.8 | 99.7 |
| 19 | 0.051 | 5.4 | 99.7 |
| 20 | 0.012 | 1.4 | 99.8 |
| 22 | 0.014 | 1.4 | 100 |
| 25 | 0.000 | 0.0 | 100 |

Table S11. Threshold values of original FINDRISC in male group

| Threshold values | Youden' index | Sensitivity (%) | Specificity (%) |
| --- | --- | --- | --- |
| -1 | 0.000 | 100.0 | 0.0 |
| 1 | 0.047 | 89.7 | 15.1 |
| 2 | 0.083 | 89.7 | 18.7 |
| 3 | 0.092 | 75.9 | 33.3 |
| 4 | 0.146 | 69.0 | 45.6 |
| 5 | 0.195 | 65.5 | 54.0 |
| 6 | 0.167 | 55.2 | 61.5 |
| 7 | 0.167 | 44.8 | 71.8 |
| 8 | 0.107 | 34.5 | 76.2 |
| 9 | 0.167 | 31.0 | 85.7 |
| 10 | 0.203 | 31.0 | 89.3 |
| 11 | 0.235 | 31.0 | 92.5 |
| 12 | 0.206 | 24.1 | 96.4 |
| 13 | 0.210 | 24.1 | 96.8 |
| 14 | 0.183 | 20.7 | 97.6 |
| 15 | 0.153 | 17.2 | 98.0 |
| 16 | 0.096 | 10.3 | 99.2 |
| 17 | 0.099 | 10.3 | 99.6 |
| 19 | 0.065 | 6.9 | 99.6 |
| 22 | 0.034 | 3.4 | 100.0 |
| 25 | 0.000 | 0.0 | 100.0 |

Table S12. Threshold values of original FINDRISC in female group

| Threshold values | Youden' index | Sensitivity (%) | Specificity (%) |
| --- | --- | --- | --- |
| -1 | 0.000 | 100.0 | 0.0 |
| 1 | 0.117 | 97.8 | 14.0 |
| 2 | 0.141 | 97.8 | 16.3 |
| 3 | 0.232 | 91.1 | 32.0 |
| 4 | 0.271 | 88.9 | 38.2 |
| 5 | 0.280 | 82.2 | 45.7 |
| 6 | 0.319 | 75.6 | 56.3 |
| 7 | 0.349 | 71.1 | 63.8 |
| 8 | 0.412 | 68.9 | 72.4 |
| 9 | 0.366 | 57.8 | 78.8 |
| 10 | 0.403 | 55.6 | 84.8 |
| 11 | 0.420 | 53.3 | 88.6 |
| 12 | 0.352 | 42.2 | 93.0 |
| 13 | 0.284 | 33.3 | 95.1 |
| 14 | 0.228 | 26.7 | 96.1 |
| 15 | 0.194 | 22.2 | 97.2 |
| 16 | 0.120 | 13.3 | 98.7 |
| 17 | 0.056 | 6.7 | 99.0 |
| 18 | 0.042 | 4.4 | 99.7 |
| 20 | -0.003 | 0.0 | 99.7 |
| 21 | 0.000 | 0.0 | 100.0 |

Table S13. Threshold values of MS in total population

| Threshold values | Youden' index | Sensitivity (%) | Specificity (%) |
| --- | --- | --- | --- |
| -1 | 0.000 | 100.0 | 0.0 |
| 1 | 0.139 | 97.1 | 16.8 |
| 2 | 0.157 | 95.9 | 19.8 |
| 3 | 0.321 | 93.6 | 38.4 |
| 4 | 0.382 | 90.1 | 48.1 |
| 5 | 0.397 | 83.7 | 56.0 |
| 6 | 0.466 | 79.7 | 66.9 |
| 7 | 0.508 | 74.4 | 76.3 |
| 8 | 0.458 | 64.0 | 81.9 |
| 9 | 0.399 | 51.7 | 88.2 |
| 10 | 0.338 | 42.4 | 91.3 |
| 11 | 0.313 | 37.2 | 94.1 |
| 12 | 0.246 | 27.3 | 97.2 |
| 13 | 0.208 | 22.7 | 98.2 |
| 14 | 0.173 | 18.6 | 98.7 |
| 15 | 0.134 | 14.5 | 98.9 |
| 16 | 0.062 | 7.0 | 99.3 |
| 17 | 0.033 | 4.1 | 99.3 |
| 18 | 0.033 | 3.5 | 99.8 |
| 19 | 0.027 | 2.9 | 99.8 |
| 20 | 0.012 | 1.2 | 100.0 |
| 22 | 0.006 | 0.6 | 100.0 |
| 25 | 0.000 | 0.0 | 100.0 |

Table S14. Threshold values of MS in male group

| Threshold values | Youden' index | Sensitivity (%) | Specificity (%) |
| --- | --- | --- | --- |
| -1 | 0 | 100.0 | 0.0 |
| 1 | 0.104 | 93.5 | 16.9 |
| 2 | 0.104 | 90.3 | 20.1 |
| 3 | 0.271 | 88.7 | 38.4 |
| 4 | 0.339 | 82.3 | 51.6 |
| 5 | 0.336 | 74.2 | 59.4 |
| 6 | 0.395 | 71.0 | 68.5 |
| 7 | 0.424 | 62.9 | 79.5 |
| 8 | 0.363 | 53.2 | 83.1 |
| 9 | 0.332 | 41.9 | 91.3 |
| 10 | 0.291 | 35.5 | 93.6 |
| 11 | 0.265 | 30.6 | 95.9 |
| 12 | 0.217 | 22.6 | 99.1 |
| 13 | 0.221 | 22.6 | 99.5 |
| 14 | 0.172 | 17.7 | 99.5 |
| 15 | 0.14 | 14.5 | 99.5 |
| 16 | 0.081 | 8.1 | 100.0 |
| 17 | 0.065 | 6.5 | 100.0 |
| 19 | 0.048 | 4.8 | 100.0 |
| 22 | 0.016 | 1.6 | 100.0 |
| 25 | 0 | 0.0 | 100.0 |

Table S15. Threshold values of MS in female group

| Threshold values | Youden' index | Sensitivity (%) | Specificity (%) |
| --- | --- | --- | --- |
| -1 | 0.000 | 100.0 | 0.0 |
| 1 | 0.159 | 99.1 | 16.8 |
| 2 | 0.187 | 99.1 | 19.6 |
| 3 | 0.349 | 96.4 | 38.5 |
| 4 | 0.402 | 94.5 | 45.7 |
| 5 | 0.428 | 89.1 | 53.7 |
| 6 | 0.504 | 84.5 | 65.8 |
| 7 | 0.551 | 80.9 | 74.2 |
| 8 | 0.511 | 70.0 | 81.1 |
| 9 | 0.433 | 57.3 | 86.0 |
| 10 | 0.361 | 46.4 | 89.8 |
| 11 | 0.338 | 40.9 | 92.9 |
| 12 | 0.260 | 30.0 | 96.0 |
| 13 | 0.199 | 22.7 | 97.2 |
| 14 | 0.172 | 19.1 | 98.1 |
| 15 | 0.130 | 14.5 | 98.4 |
| 16 | 0.051 | 6.4 | 98.8 |
| 17 | 0.015 | 2.7 | 98.8 |
| 18 | 0.015 | 1.8 | 99.7 |
| 20 | 0.009 | 0.9 | 100.0 |
| 21 | 0.000 | 0.0 | 100.0 |
